# Brain computed tomography perfusion analysis in HIV-seropositive adults with and without neurocognitive impairment in Nigeria: outcomes and challenges of a pilot study

**DOI:** 10.11604/pamj.2023.46.15.36320

**Published:** 2023-09-12

**Authors:** Godwin Ogbole, Richard Efidi, Joseph Odo, Chinonye Okorie, Tomiwa Makanjuola, Abiodun Adeyinka, Christina Sammet, Baiba Berzins, Akpa Onoja, Adesola Ogunniyi, Ann Ragin, Babafemi Taiwo

**Affiliations:** 1 Department of Radiology, College of Medicine, University of Ibadan, Ibadan, Nigeria; 2 Department of Radiology, University College Hospital (UCH), University of Ibadan, Ibadan, Nigeria; 3 Department of Neurology, University College Hospital (UCH), Ibadan, Nigeria; 4 Ann & Robert H. Lurie Children's Hospital Chicago, Chicago, Illinois 60611, the United States; 5 Division of Infectious Diseases, Feinberg School of Medicine, Northwestern University, Chicago, United States; 6 Department of Biostatistics, University of Ibadan, Ibadan, Nigeria; 7 Department of Radiology, Northwestern University, Evanston, Illinois, United States

**Keywords:** HIV, neurocognitive, HIV-associated neurocognitive disorder, people living with HIV, cerebrovascular, computed tomography perfusion, neuroimaging, neuroAID

## Abstract

**Introduction:**

the significance of cerebrovascular disease in HIV-associated neurocognitive disorder (HAND) in a homogeneous black population has not yet been determined. This incident case-control study used CT perfusion imaging to quantify and compare regional cerebral blood flow parameters in neuro-cognitively impaired and unimpaired HIV+ participants of the Ibadan Cohort on Neuro AIDS (ICON) in Nigeria.

**Methods:**

this was an incident case-control study consisting of twenty-seven HIV+ adults, classified based on Frascati criteria into neurocognitive impaired (n=18) and unimpaired (n=9) groups, who had brain computed tomographic perfusion (CTP) with a 64-slice Toshiba T scanner. The standard deviation (SD) of regional mean transit time (MTT), cerebral blood flow (CBF), and cerebral blood volume (CBV) values were calculated for bilateral basal ganglia (BG), frontal, parietal, temporal, and occipital regions from CT perfusion maps. The regional mean values and variability (SD) in the CTP measures were compared in the groups using an independent student t-test.

**Results:**

differentially higher variability in the bilateral CBF measures in the parietal (right; OR = 1.14, x̄ =5.61, p=0.041, CI=0.27-11.35/left; OR = 1.16, x̄=7.01, p=0.03, CI=5.6-13.47) and time to peak (TTP) measures in the basal ganglia (right; OR = 3.78, x̄=0.88, p=0.032, CI=0.081-1.67/left; OR = 2.44, x̄=1.48, p=0.020, CI=0.26-2.71) and occipital (right; OR = 2.18, x̄=1.32, p=0.018, CI=0.25-2.38/left; OR = 1.93, x̄=1.08, p=0.034, CI=0.086-2.06) regions were observed in the cognitively impaired group compared to the unimpaired group.

**Conclusion:**

the study evidence suggests that alterations in cerebral perfusion implicated in HIV-associated neurocognitive disorder may be possibly demonstrated using CTP, a readily available resource in most African countries saddled with the highest burden of HIV.

## INTRODUCTION

HIV-associated neurocognitive disorder (HAND) and other neurologic complications of HIV affect more than 2 million people living with HIV (PLWH) in Nigeria [1,2]. Despite increasing access to treatment, the prevalence of HAND continues to increase due to the longer life expectancy from the use of highly active antiretroviral therapy (HAART) [3]. In addition to HAND, PLWH in Nigeria has elevated risks for cerebrovascular disease (CVD) or stroke [4,5]. Furthermore, there is converging neuroimaging and neuropathology evidence that CVD may be a mechanistic pathway for the development of neurocognitive impairment in PLWH [6-10]. Several advanced neuroimaging techniques, including magnetic resonance (MR) perfusion, single photon emission computed tomography (SPECT), arterial spin labeling (ASL), and positron emission tomography (PET) have shown inconclusive and sometimes controversial cerebral perfusion and permeability abnormalities in HAND patients at different stages [10-17]. These recognized neuroimaging techniques are nonetheless complex and expensive with limited availability in developing countries like Nigeria [18-20].

We hypothesized that brain computed tomography perfusion (CTP) may serve as an alternative technique for evaluating cerebral hemodynamic changes, including cerebral blood flow (CBF), cerebral blood volume (CBV), and mean transit time (MTT)] in PLWH in these countries. There are few if any prior neuroimaging studies that have assessed the hemodynamic changes in Nigerian PLWH which represent a homogenous black community with a heavy burden of HIV. This study enrolled cognitively impaired and unimpaired PLWH from the observational Ibadan cohort on neuroAIDS (ICON) study in Nigeria.

We explored in a case-control pilot study, the association between cerebrovascular perfusion and HAND among Nigerian PLWH using CTP. The study objective was therefore to determine if there are significant differences in brain perfusion parameters between cognitively impaired and unimpaired PLWH. Our overarching aim was to produce insights into the wider clinical potential of CT perfusion in HIV in a low-resource setting. Perfusion differences may support the hypothesis that HAND is linked to HIV-related microvascular blood vessel injury, predisposing to ischemic stroke, and multiple vascular infarcts that result in cognitive abnormalities and vascular dementia.

## METHODS

**Study design:** this was an incident case-control study. Controls were selected from the same population set at risk (ICON-Ibadan Cohort Study of NeuroAIDS) in which the incident cases occurred and were selected, within the same time period. The neuro-cognitively impaired patients formed the cases while the neuro-cognitively unimpaired patients formed the control group. A case-control study design was employed for this pilot study for three main reasons. Firstly, neurocognitive impairment in HIV-seropositive adults is an uncommon finding in the Nigerian population. A case-control design allows us to efficiently study a small number of cases in relation to a larger group of controls.

Secondly, our pilot study was to show a proof of concept to test a hypothesis and answer our research questions without incurring excessive costs. Thirdly, it allowed us to collect data on the exposure (HIV neurovascular effects measurable by brain CT perfusion parameters) and outcome (neurocognitive impairment) at a specific point in time. We identified cases and matched them with appropriate controls within the same cohort, hence minimizing any potential harm/stigma to participants. Since this was a preliminary investigative study, a case-control design provided an opportunity for quicker results since data collection from cases and controls happened simultaneously.

**Participants and setting:** twenty-seven adult PLWH aged at least 21 years; consisting of 18 neurocognitive impaired (cases) and 9 unimpaired (controls) patients (classified based on Frascati criteria) were enrolled in the project. These study participants were recruited from ICON, an observational study constituting a well-characterized clinical cohort for biomarker, pharmacologic, genomics, interventional, and other studies of NeuroAIDS. The selection of patients was based on random numbers of eligible participants from within the ICON study. All participants enrolled were on anti-retroviral therapy (ART) treatment provided informed consent and completed neuropsychological assessments. The study extended from July 2016 to February 2017 and included a subset of the ICON cohort who completed CTP imaging studies. Individuals with serum creatinine > 1.2 mg/dL, cerebrovascular risk factors (such as diabetes, hypertension, coagulopathies, smoking, and peripheral vascular disease), alcohol and other substance abuse, active opportunistic infections, and contraindications to contrast CT were excluded to minimize confounding.

**Neurocognitive assessment (case and control ascertainment):** the neuropsychological instruments employed in the ICON study included the wide range achievement test (WRAT), 4 reading test, Hopkins verbal learning test - revised (HVLT-R), trail making A and B, grooved pegboard - dominant and non-dominant, Wechsler Adult Intelligence Scale (WAIS)-III digit symbol, WAIS-III symbol search, Stroop interference task, verbal fluency (letter and category), and International HIV dementia scale. These tools are validated and have been used widely in PLWH. Participants were classified into 2 groups those with versus without neurocognitive impairment using the Frascati criteria [21]. All tests were performed by trained clinical psychometricians under the supervision of a clinical neuropsychologist in order to minimize measurement bias.

**Neuroimaging:** participants had serum creatinine assessment within 48-72 hours of the scheduled scan and only those with creatinine < 1.2 mg/dL were eligible. Participants took nothing but clear liquids after midnight before the scan and nothing orally four hours prior to the examination. All participants were scanned with a Toshiba Aquillion 64 CT scanner using a protocol adapted from Northwestern Medicine Central DuPage Hospital for cerebrovascular evaluation. This consisted of 2 series obtained in the following order: 1) non-contrast head CT from the base of the skull (C1 level) to the vertex; 2) a CT perfusion scan was performed by obtaining a 32mm-slab at a level above the circle of Willis (COW) around the basal ganglia.

**Non contrast computed tomography:** the scan of the whole brain was performed using an axial mode with no contrast enhancement using: 4mm Slice thickness, axial volume rotation time of 1.5 seconds, kVp of 120, mA of 240, with no SURE exposure, scan FOV 240mm. No breath-hold or prep-delay needed and an estimated CTDI-vol. of 75mGy.

**Computed tomography perfusion imaging:** following the unenhanced head CT, CT perfusion was performed (80 kV, 50 mA) at a region between the circle of Willis to a level 2-3cm above the lateral ventricles. A 40-50ml low osmolar contrast material (iohexol, omnipaque 350 mg I/mL) was injected at 4-5 ml/s, followed by 25ml saline “chaser” administered immediately. Scan parameters included; slice thickness = 8.0mm x 4, rotation time = 1.5, range = 32.0, D-FOV = 220-240, effective mAs = <75mA, scan time = 55-75 seconds, focus = small, maximum exposure time was < 110.0 seconds. The estimated CTDI_vol_ dose was kept below 200 mGy for all studies. To achieve excellent arterial enhancement, a region-of-interest (ROI) was selected by the technologist along the anterior cerebral artery and enhancement was plotted against time. For each patient, a unique TTP was determined. This was majorly achieved by an addition of about 6-8 seconds to the usual standard delay for contrast injection creating a total delay of about 15 seconds before scans were initiated.

**Figure 1:**
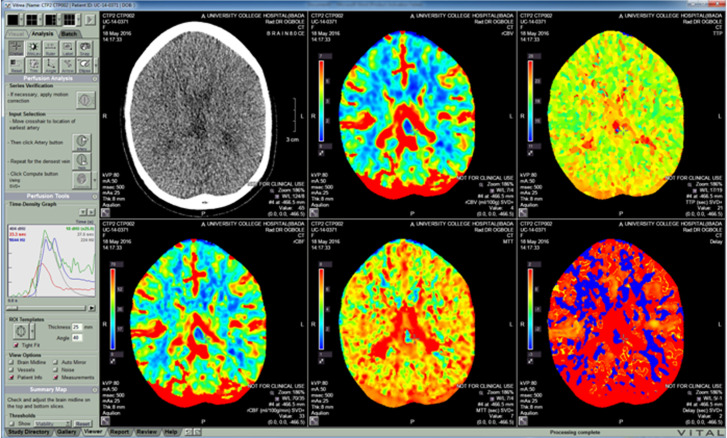
computed tomography perfusion maps on the Vitrea workstation

**Data sources/measurement/variables:** image post-processing was performed using a Vitrea v6.7 workstation (Vital Images, Minnetonka, Minnesota) with a research option to export the perfusion values of the automated region-of-interest (ROI) templates as CSV files for further processing and analysis. A ROI was applied at the appropriate slice level in the brain for neuroradiological review: cortical areas (frontal, visual cortex,), and deep grey matter (putamen, globus pallidus) Figure 1. The mean values and regional variability measurements obtained from the standard deviation values of the perfusion parameters in each of these regions of interest were reported using the following perfusion parameters: cerebral blood flow (CBF), cerebral blood volume (CBV), time to peak (TTP) and mean transit time (MTT). cerebral blood flow is the volume of blood (in mls) passing through 100g of brain tissue per minute. Cerebral blood volume is the volume of blood (in mls) in 100g of brain tissue. Mean transit time is the average period of time (in seconds) that blood spends within a determined volume of capillary circulation Time to peak is the time (in seconds) at which contrast concentration reaches its maximum. Maximum intensity projection (MIP) imaging slices were obtained at the level of the circle of Willis in orthogonal planes that incorporate all the three major cerebral vessels.

**Control of bias:** the participants were randomly selected (based on random numbers of eligible participants) from within the ICON study to minimize selection bias. All neuro-cognitive assessment tests, to classify each participant as a case or control, were performed by trained clinical psychometricians under the supervision of a clinical neuropsychologist in order to minimize measurement bias. All image analysis was conducted by reviewers blinded to participant neuro-cognitive status and other clinical and laboratory data, in order to prevent observation bias.

**Study size:** the study was conservatively sampled as a pilot to have 20 with neurocognitive impairment participants at baseline and 20 with no evidence of neurocognitive impairment, given alpha=0.05, this would provide power ≥ 0.8 to detect bivariate Pearson correlations of approximately 0.38 or greater between perfusion parameters and clinical and cognitive performance measures and establish feasibility (in vivo perfusion parameter derivation). In addition, the quantitative perfusion measures can be calculated for different brain regions in an individual subject, affording various analytic methods to augment power and reduce type 2 errors for meaningful group comparisons. This sample size is also sufficient to detect directional differences in perfusion parameters, assuming effect sizes of approximately 0.8, as defined by Cohen's [22]:


$$d=M_{1}-M_{2}/S_{pooled}$$

Where:


$$S_{pooled}=\surd \left[\left(S_{1}{}^{2}+S_{2}{}^{2}\right)/2\right]$$

**Image/quantitative variable analysis:** all image analysis was conducted at a Vitrea computer workstation with reviewers blinded to participant status and other clinical and laboratory data, in order to prevent observation bias. The Vitrea workstation was used to calculate cerebral blood volume (CBV), cerebral blood flow (CBF), mean transit time (MTT), and time to peak (TTP) measured by superimposing the ROI over each perfusion map. Anatomical regional template maps of the brain were generated and calculated mean values of each region were compared between the two groups. Motion correction and deconvolution algorithms were employed to achieve uniformity and minimize variability (Figure 1).

**Statistical analysis:** IBM SPSS version 23 (IBM SPSS Corporation, New York, USA) was used for statistical analysis with all tests being two-tailed and a p-value of < 0.05 being considered as significant. Group comparison of categorical variables (such as gender) was done using the Chi-square test. Being a case-control study, the odds ratio (OR) was computed to measure the risk/odds of neuro-cognitive impairment based on the changes in the CT perfusion parameters, in order to provide additional insights into the strength and direction of the observed associations. Group comparisons of perfusion measurements were done using an independent student t-test, in order to determine any significant difference between the neurocognitively-impaired cases and controls. Comparisons of continuous variables (such as age and CD4+ cell counts) were also done using an independent student t-test. Correlation coefficients were used to examine the relationship between baseline CD4+ T cell count and perfusion measurements.

**Ethics statement:** the study was approved by an Institution Review Committee no. UI/EC/16/0024 on 09/03/2016. Prior to enrollment, all participants provided written and informed consent to be included in the project. Unique identification (ID) numbers were assigned to each consenting participant to ensure confidentiality throughout the study.

**Source of funding:** the National Institutes of Health grant supported the study and investigators; R25 NS080949 NIH/NINDS; D43TW009608.

## RESULTS

**Participant characteristics:** there were 18 neurocognitive impaired and 9 control participants recruited into the study. There were no differences between the groups with respect to age, history of HIV or medication use, and initial laboratory findings except for gender. The cognitively impaired participants had lower recent CD4+ T cell count compared with controls, but this difference was not statistically significant (Table 1).

**Table 1:** characteristics of cognitively impaired participants and controls

	Impaired (N=18)	Control (N=9)	P-value
Age	43.2(10.9)	38.7(9.9)	0.307
Sex (male)%	46.2	12.5	0.021
Baseline CD4	193.4(183.8)	277.0(190.7)	0.297
Latest CD4	350.2(216.6)	381.6(179.2)	0.713

**Comparison of brain computed tomography perfusion measurements between people living with HIV with neurocognitive impairment and the control group:** compared to the control group, cognitively impaired participants demonstrated differentially higher variability in the bilateral CBF measures in the parietal (right; OR = 1.14, x̄ =5.61, p=0.041, CI=0.27-11.35/left; OR = 1.16, x̄=7.01, p=0.03, CI=5.6-13.47) and TTP measures in the basal ganglia (right; OR = 3.78, x̄=0.88,p=0.032, CI=0.081-1.67/left; OR = 2.44, x̄=1.48,p=0.020, CI=0.26-2.71) and occipital (right; OR = 2.18, x̄=1.32, p=0.018, CI=0.25-2.38/left; OR = 1.93, x̄=1.08, p=0.034, CI=0.086-2.06) regions compared to the unimpaired group (Table 2). We also compared the brain CT perfusion measurements in participants with neurocognitive impairment between their right and the left cerebral hemispheres and found that the right parietal and temporal regions demonstrated significantly lower variability in CBF (OR = 0.98, x̄ = -1.40, p=0.021, CI= -2.56 - -0.24) and TTP (OR = 0.86, x̄ = -0.65, p=0.036, CI= -1.245 - -0.049) measurements respectively compared to the left sides. Conversely, the right parietal region showed significantly higher variability in MTT (OR = 1.19, x̄ = 0.039, p=0.022, CI= 0.006 - 0.071) measurements compared to the left side (Table 3).

**Table 2:** areas of significant mean differences in regional variability of brain computed tomography perfusion parameters between cognitively impaired participants and controls

Parameter	Odds ratio	Mean difference	P-value	95% confidence intervals	
Right CBF parietal region	1.137	5.81	0.041	0.27	11.35
Left CBF parietal region	1.164	7.01	0.03	0.56	13.47
Right TTP occipital region	2.176	1.32	0.18	0.25	2.38
Left TTP occipital	1.934	1.08	0.034	0.086	2.06
Right TTP basal ganglia	3.784	0.88	0.032	0.081	1.67
Left TTP basal ganglia	2.435	1.48	0.020	0.26	2.71
CBF: cerebral blood flow, TTP: time to peak					

**Table 3:** difference in brain computed tomography perfusion parameters between the right and the left sides of cognitively impaired participants

Parameter	Odds ratio	Mean of paired difference between right and left sides	P-value	95% confidence intervals	
Right/left CBF parietal	0.979	-1.40	0.021	-2.56	-0.24
Right/left MTT parietal region	1.189	0.039	0.022	0.006	0.071
Right/left TTP temporal	0.859	-0.65	0.036	-1.245	-0.049
CBF: cerebral blood flow, TTP: time to peak, TTP: mean transit time					

**Correlations of computed tomography perfusion measurements with the immunologic data:** there were no significant correlations between CBV, CBF, MTT values, and baseline CD4+ T cell count (P>0.05).

## DISCUSSION

In this pilot study, we assessed cerebral perfusion changes using CTP in adult PLWH with and without neurocognitive impairment enrolled in the Ibadan cohort on neuroAIDS (ICON), study in Nigeria. We found differentially higher flow variability in the bilateral CBF measures in the parietal lobes and TTP measures in both basal ganglia of the cognitively impaired participants compared to the control group. The TTP measures of the left occipital lobe also showed increased flow variability based on the Hounsfield unit (HU) measurements Evaluating interhemispheric differences, we found that the left parietal and temporal regions demonstrated significantly (P<0.05) higher variability in CBF than the right in those with neurocognitive impairment. The clinical utility of this finding is not clearly apparent but represents early changes in the middle cerebral artery territory which is most implicated in cerebrovascular disease. The studied groups were neurologically asymptomatic. There were no significant differences between the neurocognitive impaired group and the controls in their clinical status and age. We found increased variability in the values of CBF measurements in the cortical regions of the parietal lobes of cases compared to controls. This variability may suggest flow disparities and possible microvascular infarctions in the large cortical areas of the parietal lobe which may represent CNS injury due to HIV.

Only a few studies, mostly among Caucasians have provided evidence of early cerebral perfusion alterations in neurologically asymptomatic HIV patients [13,23,24]. The recognized neurological association of HIV infection remains an important aspect of the infection and has been extensively studied with various imaging techniques with a view to unraveling the mechanistic pathways and cerebral hemodynamic determinants. We were unaware of previous studies that have employed CTP for such evaluations. While objective tests of neurological function and the use of HAART, have greatly shown clinical improvements in patients with HAND, the extent of the interrelationship between the cerebrovascular imaging parameters continues to be investigated [6,24-26]. Antiretroviral therapy effects on intracranial small vessel walls may be evaluated using noninvasive vascular imaging techniques which could serve as an effective in vivo biomarker for monitoring disease outcomes or progression in PLWH.

It remains unclear the pathologic mechanisms of spread from subcortical to cortical regions of the brain. De Alwis *et al*. [27] observed that HIV as a disease entity caused not only intracranial vessel wall thinning but also loss of vascular plasticity. These alterations cause an expansion of the vessel lumen and worsen the regulation of perfusion to subcortical and cortical cerebral regions. Some studies have shown a connection between these vascular changes and the development of HAND [28-34]. The clinical changes in HAND are usually subtle and progress over a long period, making it challenging to study the imaging changes as well.

**Study limitations:** this study had a number of limitations. Although adequately planned overall, the small sample examined fell short of the intended number of twenty in each group for the pilot as the equipment employed for the study had several long intermittent downtimes resulting in dropouts of eligible participants. The low number of controls for HAND subjects may be a reason for the reduced difference in both previously identified regions of the brain for HAND patients. The impaired group had a higher proportion of females than the control group, so possible gender variation in the groups cannot be completely excluded. Previous studies have shown that cerebrovascular risk factors and cognitive changes have gender influences and associations. The results in those studies could have been affected by differences in traditional cardiovascular risk factor profiles between the gender groups (despite being adjusted for statistically) [35,36].

The attempt to use matching data for age and sex in our pilot study could have helped reduce the effect of gender disparity in our results. However, this was not the case. The small number of eligible participants did not allow room for sub-classifications of HAND patients, as we know that severity is related to several other factors. We only grouped all into a batch group of “cognitively impaired” which understandably has subclasses. Our aim was to establish any significant difference between the two groups based on a possible differential in cerebral blood flow. Since these patients presumably already had compromised cerebral blood flow due to HIV infection, there was the need to establish a reference point for each patient. The cerebellum was the best baseline parameter to use as a control for each candidate and to determine a proportionate difference in cerebral flow if it existed since it is rarely affected by HIV. The difference in the ratio would have provided a good basis for comparisons among the with/without groups with cognitive impairment. Unfortunately, we could not obtain blood flow parameters for the cerebellum in all patients. We only targeted the region of the basal ganglia around the circle of Willis since our machine did not have the capability to do a full brain perfusion including the cerebellum.

## CONCLUSION

In this pilot brain CT Perfusion study among black Africans using the most widely available cranial imaging system, we demonstrate the likelihood of vascular dysfunction in subcortical and cortical areas of the brain in HAND patients on treatment compared with controls. This pilot study has provided some evidence of differential cerebral perfusion changes among treated PLWH with measurable cognitive impairment. Despite the use of HAART and being asymptomatic for HAND, there were detectable cerebral vascular changes that could predispose to cerebrovascular events and hence require closer observation. Cerebral blood flow abnormalities and vascular disruptions have been found to be independently predictive of future stroke and the development of HAND in PLWH [6,9,26]. It is known that multiple mechanisms are involved in the development of HAND, nevertheless, current indications suggest a high contribution of inflammation and other vascular network dysfunction.

Improving our understanding of the multiple etiologic channels among Nigerians may assist in developing potent and rationally affordable interventions in places of low resources such as Africa. This pilot evaluation of imaging perfusion parameters provided some insights into the challenges of evaluating cerebral blood flow abnormalities using CTP. Nevertheless, it also provides an opportunity for the introduction of CTP for cerebral hemodynamic evaluations and possibly in the management of acute stroke in Nigeria as CT is a more widely available modality than Magnetic Resonance Imaging (MRI) for perfusion studies.

Future studies using more robust perfusion techniques will need to evaluate longitudinal imaging changes in HAND to characterize the potential contribution of comorbid conditions as well as to determine whether imaging biomarkers of cerebral injury are associated with changes in the clinical characteristics of HAND in African patients.

### What is known about this topic

- *Advanced neuro-imaging techniques, including MR Perfusion, single photon emission computed tomography (SPECT), Arterial Spin Labeling (ASL), and Positron Emission Tomography (PET) have been used to assess cerebral perfusion changes in HAND patients in predominantly Caucasian populations;*
- *There is evidence of early cerebral perfusion alterations/abnormalities in neurologically asymptomatic HIV patients*.

### What this study adds

- *The current study demonstrated the use of CT perfusion technique and differences in the cerebral perfusion parameters in a homogeneous cohort of black individuals with HIV with and without cognitive impairment;*
- *The current study may provide opportunities for the introduction of CT perfusion for cerebral hemodynamic evaluations in acute stroke management in African regions like Nigeria since CT is more widely available than MRI for perfusion studies in most of Africa;*
- *The current study represents the first attempt to use CTP for assessing cerebral perfusion changes in HAND patients in a homogenous African population*.
